# Triple therapy: Three departments collaborating to train medical students in rural settings

**DOI:** 10.4102/phcfm.v16i1.4553

**Published:** 2024-07-11

**Authors:** Francois Coetzee, Maria E. van Zyl, Maryke Geldenhuys, Kobus Viljoen

**Affiliations:** 1 Ukwanda Centre for Rural Health, Faculty of Medicine and Health Sciences, Stellenbosch University, Cape Town, South Africa; 2 Division of Disability and Rehabilitation Studies, Faculty of Medicine and Health Sciences, Stellenbosch University, Cape Town, South Africa; 3 Division of Health Systems and Public Health, Faculty of Medicine and Health Sciences, Stellenbosch University, Cape Town, South Africa; 4 Department of Family and Emergency Medicine, Faculty of Medicine and Health Sciences, Stellenbosch University, Cape Town, South Africa

**Keywords:** primary healthcare, undergraduate training, rural hospitals, interprofessional collaboration, service learning, community-based learning

## Abstract

The primary healthcare (PHC) rotation places medical students in rural district hospitals for 4 weeks during their 4th or 5th year. This rotation is a collaboration among three academic units at Stellenbosch University’s Faculty of Medicine and Health Sciences. Learning activities during this rotation include participation in a longitudinal community-oriented primary care project, conducting rehabilitation-oriented home visits to persons with disabilities, and assessing and treating patients presenting with undifferentiated problems on an in- and outpatient basis. Working in rural contexts for a month affords students opportunities to foster meaningful relationships with the healthcare team, patients and the community, while learning about collaborative teamwork and communities. Critical reflections about the interprofessional care of patients and a community evaluation are key components of the students’ learning and assessment. Demonstrating the importance of interprofessional collaboration in PHC, this integrated training model has received, and continues to receive, positive feedback from students and the clinicians involved. Attention to logistics and academic support plays a crucial role in ensuring optimal learning for students. An integrated approach that involves multiple academic units, various healthcare professions and communities is strongly recommended for those who are considering training students in rural PHC environments.

## Background

Undergraduate training of medical students should include a significant portion of primary healthcare (PHC) training in community settings where students can experience continuity of care and get a better understanding of community contexts’ influence on health-related issues.^1,2,3^ It is suggested that such training be integrated by including training in public health to increase medical students’ social accountability.^4^

## The characteristics of the primary healthcare rotation

The PHC rotation is an integrated rotation, meaning it is jointly managed by three academic units within the Stellenbosch University Faculty of Medicine and Health Sciences (FMHS): Family and Emergency Medicine, Health Systems and Public Health, Disability and Rehabilitation studies. The rotation places 4th or 5th year medical students at rural district hospitals in the Western Cape or Northern Cape for 4 weeks.

## The learning outcomes for the primary healthcare rotation

Through sustained collaboration, the three academic units have synthesised a set of learning outcomes that are integrated and shared across the learning activities and assessments. Integrating the learning activities and assessments led to more patient-learning time for students and a decrease in the assessment burden for clinicians and students. The learning outcomes for the PHC rotation are listed below.

At the end of the rotation, students should be able to:

- use clinical practice guidelines while applying the principles of evidence-based medicine
- connect and understand how the health problems of the patients they encounter are influenced by the patients’ contexts
- describe the community oriented primary care (COPC) process and apply it to propose solutions to community health problems
- communicate effectively during consultations
- perform a thorough 3-stage assessment and develop an inter-professional management plan for patients presenting with common problems in primary care or district hospital settings
- critically reflect on the impact of ill health and/or disability on individuals, families, caregivers and the immediate community
- perform a range of common procedural skills in primary care or district hospital settings.

## The context and the learning activities

Students are placed in groups of 2 to 4 at one of the 18 rural primary healthcare environments within the district health system of the Western Cape and Northern Cape provinces. Here they are integrated into the clinical teams by active participation in the care of patients, under the clinical supervision of either a Family Physician, a Medical Officer or Community Service doctor as well as a PHC Facilitator, who is usually an allied health professional. In addition, students have to engage with patients and communities outside of the formal healthcare setting, when doing contextual visits at patients’ homes or their places of employment and when visiting non-governmental organisations. These organisations include old age homes, addiction centres, schools and early childhood development centres.

Being embedded in a rural context benefits students’ learning in several ways. Apart from providing ample opportunities for honing clinical and procedural skills, rural contexts support meaningful learning relationships with patients and preceptors.^5^ It supports interprofessional and transformative learning,^6,7^ and the contributions students make through their longitudinal COPC projects are valued by staff members in rural environments.^2^ During the 4-week rotation, students also have ample learning opportunities while working alongside health professionals, including occupational therapists, physiotherapists, dieticians, social workers, nursing staff, audiologists and community care workers. Students must complete several tasks during their training, which include:

- A longitudinal COPC project in the community they have been placed in; students, in collaboration with relevant stakeholders, define and measure a problem at the community level and then develop, implement and monitor interventions to address the identified problems.
- Comprehensive patient assessments; students use the World Health Organization’s (WHO) International Classification of Functioning and Disability to holistically assess two patients with disability to develop and initiate the implementation of interprofessional management plans.
- Completing a logbook of 10 patient presentations to health professionals; two of them should be observed consultations using the mini-CEX assessment tool.^8^
- Completing a logbook of at least 20 procedures; a specified list is provided in the study guide.
- Completing 38 h of on-call duties in an emergency centre.
- Participation in online discussion forums; students are required to make an original contribution and respond to fellow students’ contributions.

## Assessment for learning

The premise of assessment for learning is that the assessment programme generates rich information that will support individual students in their learning.^9,10^ The assessments in the PHC module assess the students’ knowledge and competencies for all three units in joint assessments conducted by the clinical supervisor and PHC facilitator as opposed to separate assessments for each unit and provides personalised feedback to each student. The personalised feedback is made possible by involving clinicians at the rural district hospital as examiners in the interview assessment of students at the end of each rotation. The local clinicians are also involved with the signing off of the logbooks and calls, and the assessment of the COPC project. The assessments are situated within a competency-based education framework and care was taken to ensure constructive alignment of the learning outcomes, the learning activities and assessments.^11^ See Table 1 for a summary of the PHC module assessments and the contributions of each assessment to the module mark.

**TABLE 1:** The assessments in the primary healthcare rotation.

Assessment	Format	Contribution (%)
International Classification of Function interprofessional management plan	Online submission	10
Online critical self-reflection and peer feedback	Online submission	30
COPC project and online activities	Group presentation	30
Individual interview (includes a comprehensive patient management interview and verification of completion of logbook tasks)	Interview	30
**Total**		**100**

COPC, community oriented primary care.

An assessment programme in the context of competency-based education should include continuous assessment, formative assessment, summative assessment, and assessments that take place in the context of the workplace environment.^12^ This is true in the PHC module and the presence of these elements of assessment is also aligned with the requirements of the Health Professions Council of South Africa, which emphasises social accountability and the development of the graduate competencies of healthcare professionals.^13^

## Student support

It is crucial to provide excellent academic and logistic support to students. As the number of students increased on the distributed training platform, in order to place students from five training programmes at almost 80 different training sites, the need for logistic support increased and in 2017 a logistics unit, Stellenbosch University Logistics for the Clinical Training Platform (SUNLOC), was created within the FMHS. SUNLOC provides students with accommodation, transport, and information and communication technology services (ICT) and has played a foundational role in the success of this integrated rotation.

## Rolling with rural realities

Placing students in groups of two to four in rural environments does come with challenges. Students are often confronted by the limited medical resources in their context, as well as the relative social isolation and typical rural infrastructure challenges like navigating dirt roads, limited shopping options, having erratic water and electricity supply or unreliable internet access. However, from informal feedback students seem to relish the adventure it represents and almost all the students report an overall positive experience at the end of the rotation.

For the academic coordinators, challenges relate to faculty development of the supervising doctors because of the high staff turnover that some sites experience and to ensure that all the training sites meet a certain minimum set of criteria that will allow students to meet their learning outcomes. Annual site visits have played a vital role in dealing with these challenges and making certain that the three academic units maintain alignment.

## Feedback from the students

Despite working hard, students frequently comment on how invigorating the rural learning experience is. Aspects frequently mentioned by students are the abundance of opportunities to hone clinical skills,^14^ the transformative experience of conducting home visits,^7^ and the overall ‘niceness’ of rural clinicians are frequently mentioned. Students often comment that they ‘feel liked’, ‘feel useful’ and ‘have learnt so much’. The mentioned experiences contribute towards nurturing a healthy professional identity.^15^ Students are able to observe the interdependent nature of healthcare in rural settings and often reflect on how they have gained a better understanding of the significant roles of allied health professionals in the care of patients. Students often find bridging the difference between the ‘purist’ tertiary training context, and the ‘pragmatic’ rural context a challenge. Management plans often have to be context-adapted, which occasionally creates uncertainty in the minds of those still trying to cement the foundations. However, seeing a different way of ‘doing things’ allows for critical reflections about the care of patients and may motivate students to expand their knowledge regarding the correct treatment for a condition.^6^

## Our learning journey

This module has been offering rural learning experiences to undergraduate students for more than 25 years, and during this time we have learned important lessons, which include:

- Time spent to achieve a shared vision and an integrated assessment strategy for the rotation proved to be of critical importance. Integration of learning and integration into teams are not spontaneous processes; it takes time, and is dependent on good relationships.
- Excellent collaboration and early communication with those involved with logistics is non-negotiable.
- Regular site visits are an important strategy to build relationships, deal with logistics issues and support training sites.
- A focused agenda is needed for site visits, and it should include assessment support and faculty development for the clinician-preceptors.

## Conclusion

The integrated PHC module offers medical students an opportunity to experience rural healthcare environments and interprofessional collaboration that involves rehabilitation professionals and key stakeholders in the community. Students are afforded opportunities to gain a wider perspective than in-hospital care and can engage with patients in the context of their homes and their community, leading to a greater understanding of the social determinants of health and the challenges that patients experience in accessing care.

By combining the learning outcomes, activities, and assessments of three academic units, we foster collaboration that demonstrate to students how to bridge fragmented care in the healthcare system, promoting integrated approaches that break down silos and enhance patient care. The district health system is very reliant on interprofessional teams, collaboration and good collegial relationships, which supports an integrated approach to training medical students in PHC. We strongly recommend an integrated PHC rotation that incorporates the public health and rehabilitation aspects of PHC as it is positively experienced by students and their preceptors.

## References

[1] Essuman A, Mash R, Besigye I, Flinkenflögel M. Conference report: Undergraduate family medicine and primary care training in Sub-Saharan Africa: Reflections of the PRIMAFAMED network. Afr J Prim Health Care Fam Med. 2017;9(1):1–5. 10.4102/phcfm.v9i1.1351 PMC5291078 28155289

[2] Reid S, Conradie H, Daniels-Felix D. The effect of undergraduate students on district health services delivery in the Western Cape Province, South Africa. Afr J Health Prof Educ. 2018;10(1):56. 10.7196/AJHPE.2018.v10i1.959

[3] Pettigrew LM, MacVicar R. Overcoming challenges in primary care education. Educ Prim Care. 2015;26(2):120–121. 10.1080/14739879.2015.11494325 25898304

[4] Punzalan JK, Guingona M, Punzalan MG, Cristobal F, Frahsa A, Liwanag HJ. The integration of primary care and public health in medical students’ training based on social accountability and community-engaged medical education. Int J Public Health. 2023;68:1605359. 10.3389/ijph.2023.1605359 36776739 PMC9908606

[5] Maley M, Worley P, Dent J. Using rural and remote settings in the undergraduate medical curriculum: AMEE Guide No. 47. Med Teach. 2009;31(11):969–983. 10.3109/01421590903111234 19909036

[6] Müller J, Reardon C, Coetzee F, et al. Transformative learning through participation: Experiences at a rural clinical training site in South Africa. BMC Med Educ. 2022;22(1):1–11. 10.1186/s12909-022-03233-w 35296325 PMC8928645

[7] Van Zyl M, Archer E. Shifting perspectives: Unveiling the transformative potential of home visits to persons living with disability in rural settings for medical students. Med Teach. 2023;(45):1–7. 10.1080/0142159X.2023.2292981 38104586

[8] Norcini JJ, Blank LL, Duffy FD, Fortna GS. Academia and clinic The Mini-CEX : A method for assessing clinical skills. Ann Intern Med. 2003;138(6):476–481. 12639081 10.7326/0003-4819-138-6-200303180-00012

[9] Schuwirth LWT, Van der Vleuten CPM. Programmatic assessment: From assessment of learning to assessment for learning. Med Teach. 2011;33(6):478–485. 10.3109/0142159X.2011.565828 21609177

[10] Swanwick T, Forrest K, O’Brien BC, editors. Understanding medical education: Evidence, theory, and practice. San Francisco: John Wiley & Sons; 2018 Oct 2.

[11] Pangaro L, Ten Cate O. Frameworks for learner assessment in medicine: AMEE Guide No. 78. Medical Teacher. 2013 Jun 1;35(6):e1197–210. 23676179 10.3109/0142159X.2013.788789

[12] Holmboe ES, Sherbino J, Long DM, Swing SR, Frank JR. The role of assessment in competency-based medical education. Medical teacher. 2010;32(8):676–82. 20662580 10.3109/0142159X.2010.500704

[13] Kalafatis N, Sommerville T, Gopalan P. Defining fitness for purpose in South African anaesthesiologists using a Delphi technique to assess the CanMEDS framework. South Afr J Anaesth Analg. 2019;25(2):7–16. 10.36303/SAJAA.2019.25.2.2193

[14] Reid S, Worley P, Strasser R, Couper I, Rourke J. What brings us together: The values and principles of rural medical education. In: Chater AB, Rourke J, Strasser R, Couper I, Reid S, editors. WONCA Rural Medical Education Guidebook. World Organization of Family Doctors (WONCA): Working Party on Rural Practice, Bangkok; 2014 Mar 20:43–58.

[15] Krishnasamy N, Hasamnis AA, Patil SS. Developing professional identity among undergraduate medical students in a competency-based curriculum: Educators’ perspective. J Educ Health Promot. 2022;11(1):361. 10.4103/jehp.jehp_329_22 36618475 PMC9818684

